# Publishing student-led discoveries in genetics

**DOI:** 10.1093/g3journal/jkac141

**Published:** 2022-06-21

**Authors:** Danielle Heller, Viknesh Sivanathan

**Affiliations:** Department of Science Education, Howard Hughes Medical Institute, Chevy Chase, MD 20815, USA; Department of Science Education, Howard Hughes Medical Institute, Chevy Chase, MD 20815, USA

**Keywords:** undergraduate research, course-based research, SEA-GENES, bacteriophage genetics

In a Mutant Screen Report in this issue, we (Heller *et al.*) report the findings of a genome-wide screen to identify novel mycobacteriophage gene products capable of inhibiting mycobacterial growth. Overexpression of the 94 genes encoded by phage Waterfoul in the host bacterium *Mycobacterium smegmatis* revealed 32 gene products capable of hindering mycobacterial growth to varying degrees; half of these gene products harbor no recognizable domains or homology to gene products of known function, offering many novel growth inhibitors for future characterization. This is the first report from the HHMI Science Education Alliance-Gene-function Exploration by a Network of Emerging Scientists (SEA-GENES) project, a new project that engages undergraduate scientists and their faculty instructors in an exploration of bacteriophage genetics within the context of undergraduate science courses.

The bacteriophage population is enormous (10^31^ estimated phage particles) and encodes a remarkably diverse set of genes, the vast majority of which cannot be assigned functions through bioinformatic methods (Hatfull 2015). Given the considerable importance of phages in environmental systems, bacterial pathogenesis, and their promise as therapeutic agents, experimentally investigating the functions of these genes is an important scientific endeavor. The Genetics Society of America (GSA) and *G3* are partnering with HHMI to disseminate the student-led discoveries of the SEA-GENES course-based research project to further advance our understanding of this largely uncharted system.

## Course-based research as a model for advancing science and science education

It is well-established that engaging undergraduate students in scientific research is a multifaceted and high-impact experience that improves their learning and their likelihood of persisting in the sciences (NASEM 2017). In the past 2 decades, the course-based research model has gained significant traction as a means to scale access to research opportunities for undergraduate students; in this model, authentic research is embedded directly within STEM courses, replacing traditional laboratory instruction with faculty-led research projects (Auchincloss *et al.* 2014). Such course-based research has the capacity to simultaneously advance science and science education, as exemplified by the Science Education Alliance-Phage Hunters Advancing Genomics and Evolutionary Science (SEA-PHAGES) project.

Supported by HHMI and led in collaboration with scientists at the University of Pittsburgh and James Madison University, SEA-PHAGES is a 2-semester course-based research project that is implemented annually by faculty instructors and cohorts of undergraduate students at >150 colleges and universities. Since its initiation in 2008, over 35,000 undergraduate students have participated in the SEA-PHAGES project, many of them as part of their introductory biology sequence. In the first semester of the SEA-PHAGES project, each student isolates and characterizes a novel actinobacteriophage from their local environment, and in the second semester, class teams produce a high-quality genome annotation for 1 or 2 of their discovered phages. Students emerge from this research experience with a strong foundation in microbiology and bioinformatics, and assessment data show a significant increase in their likelihood of persisting in the sciences as compared to their peers in traditional lab courses (Jordan *et al.* 2014; Hanauer *et al.* 2017).

While the rich educational experience of SEA-PHAGES is built through the deep investigation of 1 phage at a time, collectively, SEA-PHAGES researchers have compiled a rich dataset that has significantly broadened our understanding of phage populations and the ways they evolve (Pope *et al.* 2015, 2017; Hatfull 2020). As of this writing, SEA-PHAGES researchers have isolated over 20,400 novel bacteriophages capable of infecting diverse species of Actinobacteria; genomes for almost 4,000 of these phages have been annotated and submitted to GenBank, revealing thousands of gene families with no known functions. This genomic dataset can be readily explored by the broader community using the Actinobacteriophage Database (phagesdb.org) (Russell and Hatfull 2017) and the comparative genomics tool Phamerator (phamerator.org) (Cresawn *et al.* 2011), both of which were developed in support of the SEA-PHAGES project; likewise, the vast SEA-PHAGES archive continues to be mined by researchers for further exploration of fundamental questions of phage biology. In recent years, SEA-PHAGES lead scientist Graham Hatfull and his team have deployed many student-discovered phages in the development of therapeutic phage cocktails for the treatment of life-threatening mycobacterial infections (Dedrick *et al.* 2019; Little *et al.* 2022; Nick *et al.* 2022).

The SEA-GENES project is a natural evolution of the SEA-PHAGES project, going beyond genome annotation to the experimental exploration of the functions of phage genes in the bacterial host. Designed to be embedded within mid-level biology laboratory courses, the SEA-GENES project engages undergraduate SEA researchers in an additional 1–2 semesters of research, extending their exposure to the culture and process of science and providing experience in molecular cloning and genetics methods. It also leverages the outputs of the SEA-PHAGES project—the vast collection of bacteriophages, high-quality genome annotations, and trained student researchers—to generate a large genetic screening dataset to advance our understanding of the functions of the genes encoded by mycobacteriophages. At each participating school, teams of undergraduate students and their faculty instructors conduct a systematic exploration of one of the >2,000 unique sequenced mycobacteriophages in the SEA-PHAGES archive. Gene by gene, students employ molecular cloning techniques and reagents provided through partnership with New England Biolabs and Integrated DNA Technologies to build an arrayed genomic plasmid library. Then, they work collaboratively to screen this library in simple, accessible plate-based assays to comprehensively evaluate the effects of phage gene overexpression on mycobacterial host phenotypes such as growth and superinfection immunity. Among the thousands of uncharacterized gene families encoded in the mycobacteriophage population, these student-led screens reveal phage genes capable of conferring biologically interesting phenotypes, good candidates for further genetic dissection, protein interaction studies, or other characterization. Together, schools participating in SEA-GENES contribute data for a diverse collection of mycobacteriophages, providing good experimental coverage of the known sequence space; given the highly mosaic nature of these phage genomes, students across schools test many gene homologs as well, allowing for comparative functional analyses and further examination of functional domains or motifs. All generated plasmid libraries are archived with HHMI and available to the research community for further application and study, and the primary screening data will soon be available in an open access database.

## Communicating student-led discoveries

Course-based research is a powerful convergence of science and science education. In course-based research projects like SEA-PHAGES and SEA-GENES, students conduct research using basic techniques well-suited for novice scientists; each cohort contributes a novel research output that *en masse* form a large, discovery-rich dataset. Communicating these individual student-led discoveries in a timely manner and accessible format is important both for the advancement of science and for the development of the next generation of scientists. In the SEA-PHAGES project, faculty and students have coauthored almost 150 genome announcements published by the American Society of Microbiology in their *Microbiology Resource Announcements* journal, offering a brief, accessible format for describing student-characterized bacteriophage genomes. Similarly, the Mutant Screen Report by Heller *et al.* in this issue of *G3*, represents the first of many SEA-GENES reports that HHMI and GSA hope to share with this community, disseminating the experimental observations and molecular tools generated by undergraduate scientists in their collaborative exploration of the genes encoded by bacteriophages.
